# Supplementary material for: Complexome profiling of the Chlamydomonas psb28 mutant reveals TEF5 as an early PSII assembly factor
Source: Plant Cell. 2025 Mar 17;37(6):koaf055. doi: 10.1093/plcell/koaf055 (PMC12164590; doi:10.1093/plcell/koaf055)
Supplement: koaf055_Supplementary_Data [file koaf055_supplementary_data.zip › psb28_interactive.zip!/plots/intensity/qualityPlots/peptides/_Cre15.g635067.t1.1.html]

### Cre15.g635067.t1.1

Trivial name:   
SeqCoverage [median]: 21.10  
Mass: 87.09 kDa  
  
 

| Sequence | MissedCleavage | Mass [Da] | wtIntensity | muIntensity | Score |
| --- | --- | --- | --- | --- | --- |
| DSYLTTTVSSLALDVADAKK | 1 | 2097.08 | 1158.86 | 0.00 | 93.68 |
| HTDVAHPPPGLAAGGPATTAVEMGAYDKI | 1 | 2843.39 | 0.00 | 17083.57 | 73.21 |
| ISGGLGPAFAGLGPAAR | 0 | 1510.83 | 5513.48 | 8111.42 | 187.85 |
| LAAAALAAVQTAAADGGK | 0 | 1568.85 | 2897.41 | 16051.62 | 172.76 |
| LDFYVAPADLVHFGAAGEAAR | 0 | 2189.09 | 1634.19 | 0.00 | 43.12 |
| LITAGPDDTDAR | 0 | 1243.60 | 6555.62 | 13612.62 | 194.06 |
| NPGSSSTYAITHYFNK | 0 | 1785.83 | 1595.98 | 5401.31 | 112.72 |
| PLGVKPTTDVYPIFR | 1 | 1701.95 | 0.00 | 10545.78 | 92.27 |
| RDSYLTTTVSSLALDVADAKK | 2 | 2253.19 | 622.78 | 0.00 | 32.35 |
| RLAAAALAAVQTAAADGGK | 1 | 1724.95 | 1105.41 | 13142.20 | 104.88 |
| TADVGSSR | 0 | 791.38 | 0.00 | 3764.93 | 143.89 |
| TVGVTTEWPADAAGR | 0 | 1529.75 | 0.00 | 1286.16 | 77.06 |
| VMDLMMAR | 0 | 965.45 | 3964.72 | 9286.49 | 67.03 |
|  |  |  | 25048.4 | 98286.1 |  |
